# Supplementary material for: Augmenting bacterial similarity measures using a graph-based genome representation
Source: mSystems. 2024 Jun 28;9(7):e00497-24. doi: 10.1128/msystems.00497-24 (PMC11265277; doi:10.1128/msystems.00497-24)
Supplement: Supplemental Figures — Figures S1-S9. [file msystems.00497-24-s0001.docx]

**Augmenting Bacterial Similarity Using a Graph-Based Genome Representation**

Supplementary Figures


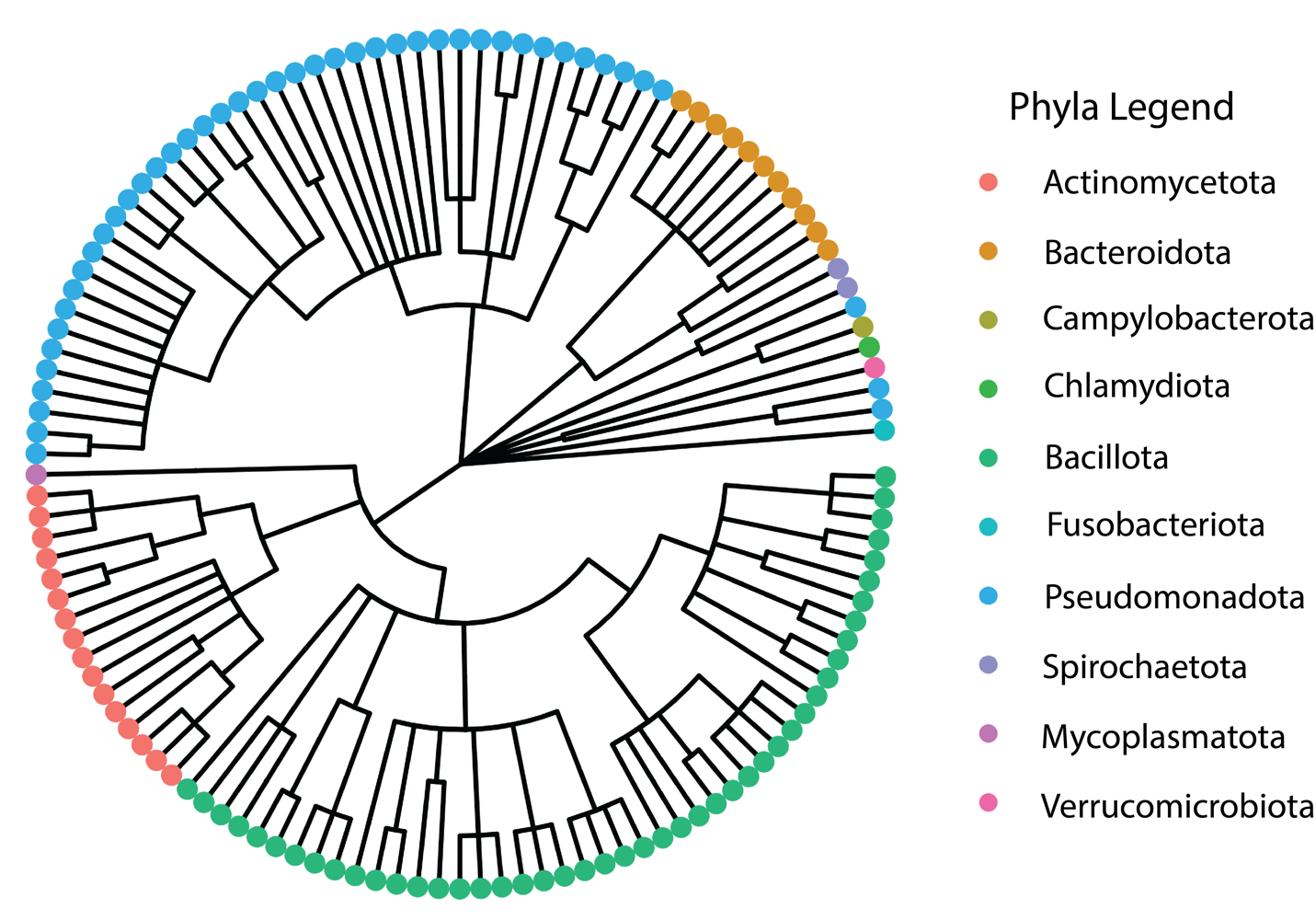
Supplementary Figure 1. Composition of GenBank genome data, representing 10 phyla of bacteria. The largest groups comprise of *Pseudomonadota, Bacillota, Actinomycetota,* and *Bacteroidota.* The phylogenetic tree structure was created with NCBI Common Tree, with the genera used for input.


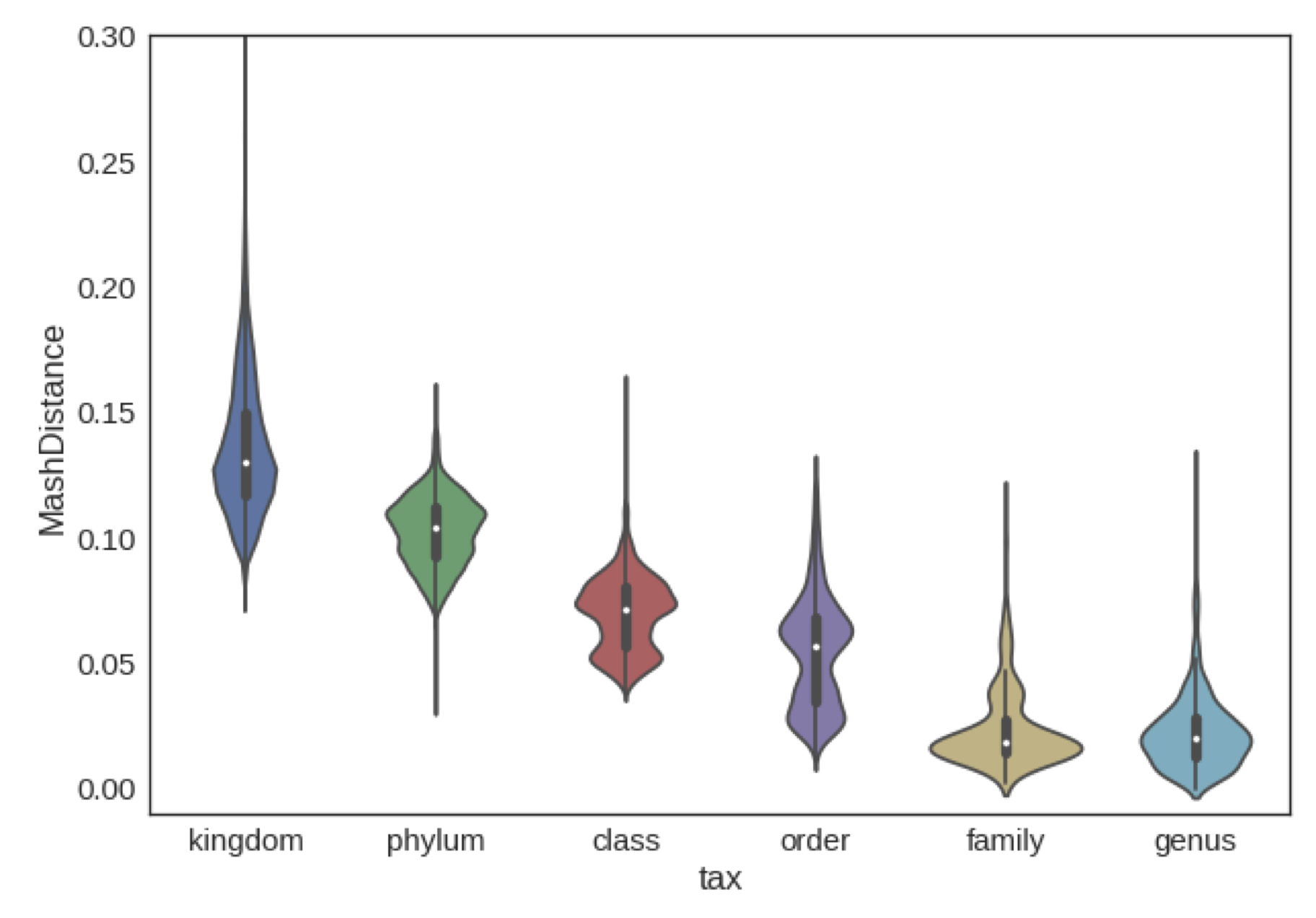


Supplementary Figure 2. Distribution of 16S rRNA MASH distances, grouped by lowest shared taxonomic group between every pair of genomes (tax). For example, if two genomes are in two different phyla, the group would be labeled as kingdom. The lowest grouping possible is genus, where two genomes would be two different species within the same genus. Mash distances of 1 are not included in the plot, as these are rough calculations based on the MASH algorithm and do not represent exact distance.


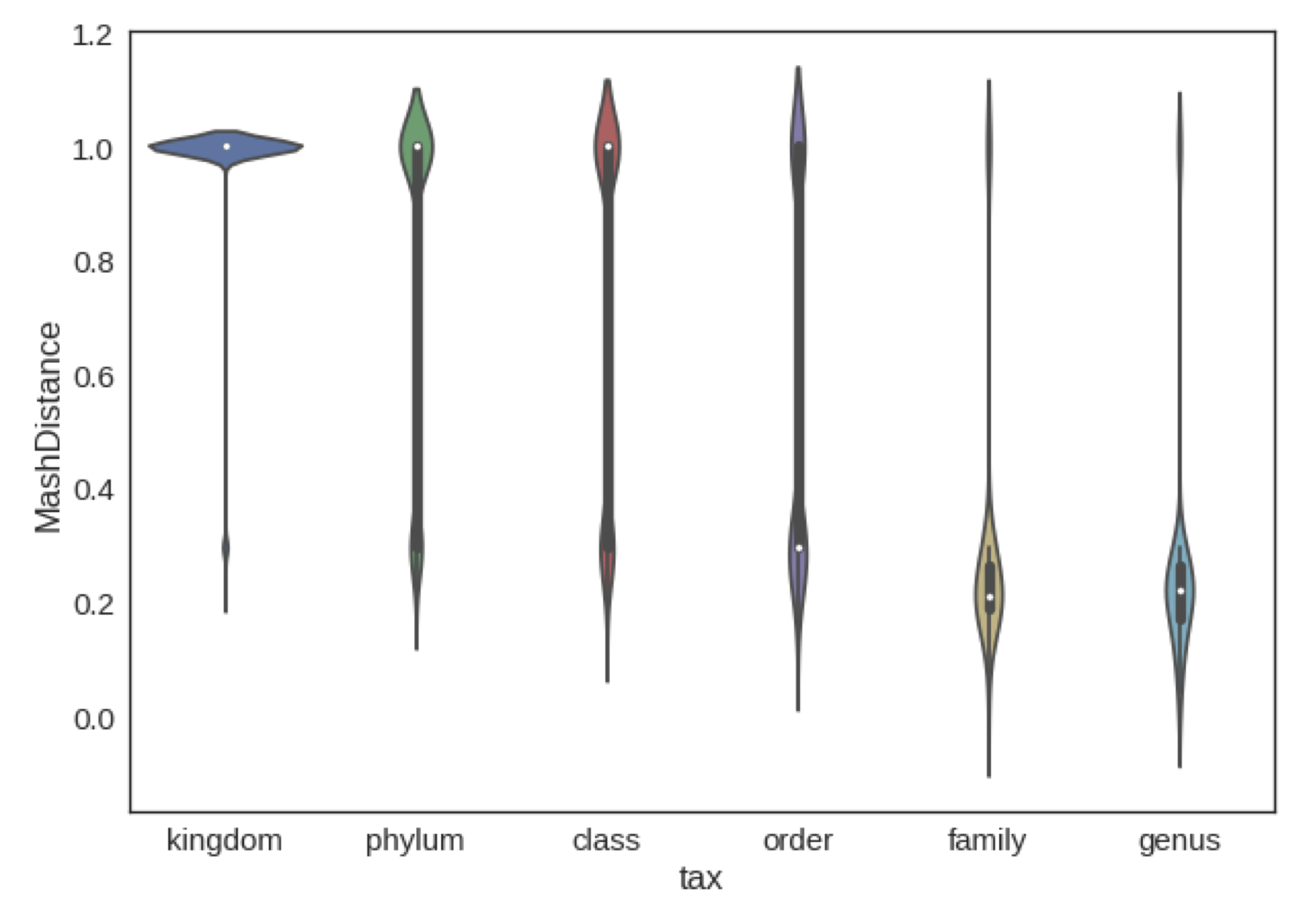


Supplementary Figure 3. Distribution of whole genome MASH distances, grouped by lowest shared taxonomic group between every pair of genomes (tax). For example, if two genomes are in two different phyla, the group would be labeled as kingdom. The lowest grouping possible is genus, where two genomes would be two different species within the same genus.


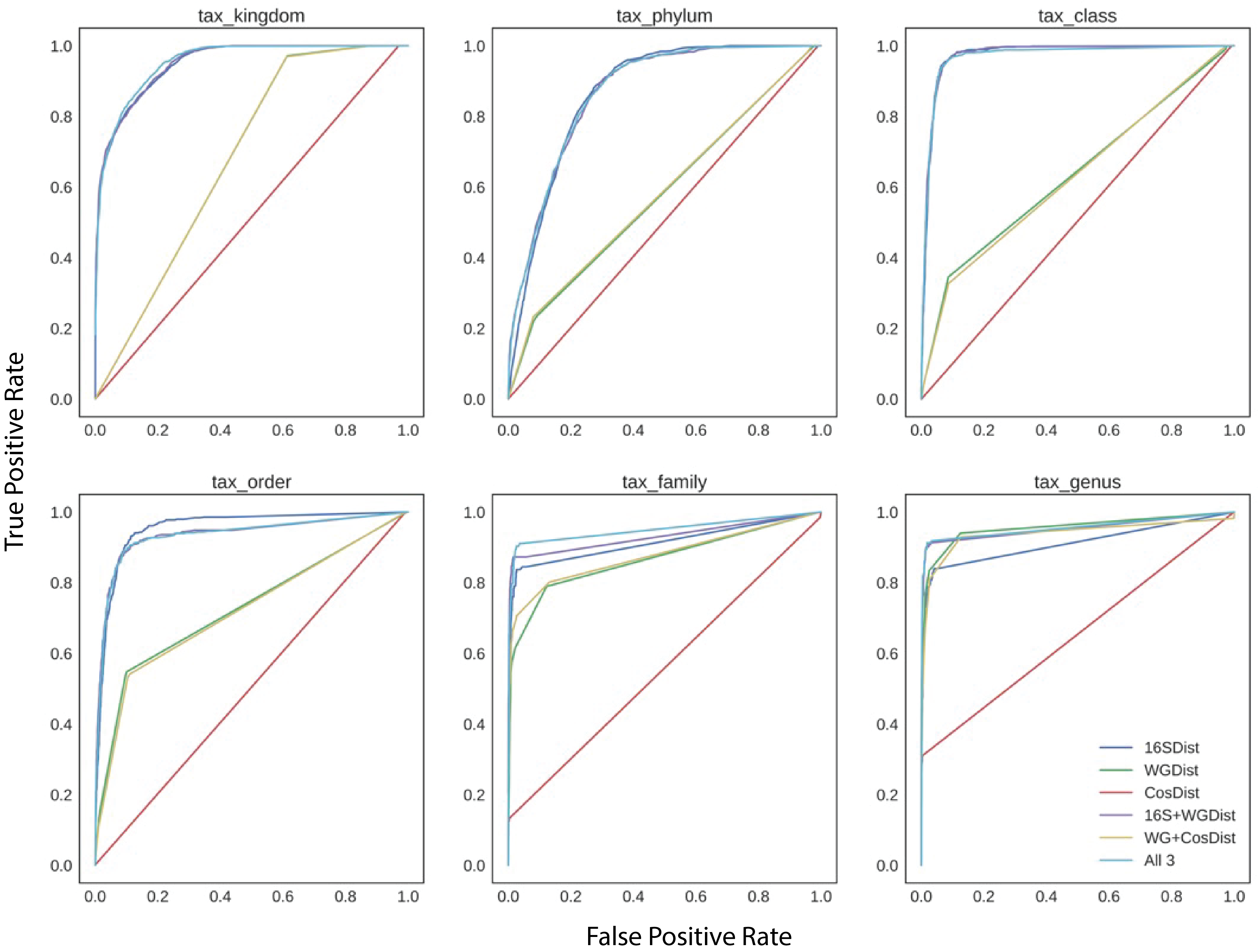


Supplementary Figure 4. The individual ROC curves for the random forest model trained on each taxonomic distance per pair (0 = pair not in the same taxonomic group, 1 = pair in the same taxonomic group). Each curve represents the data input given (16SDist: 16S MASH distance, WGDist: whole genome distance, CosDist: synteny distance).


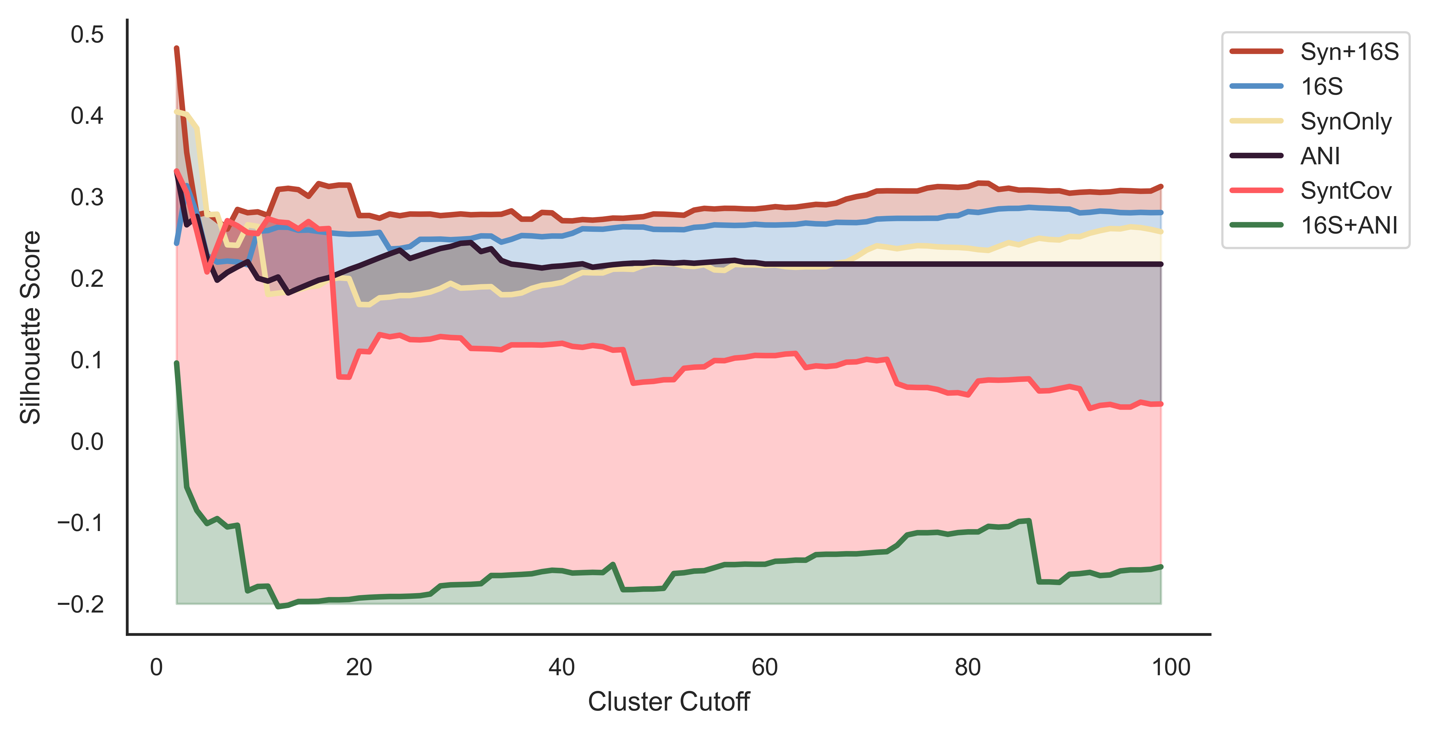


Supplementary Figure 5. Silhouette score comparison for complete-linkage hierarchical clustering. Cohort names indicate as follows - Syn+16S: the novel synteny metric added to 16S; 16S: the original 16S unchanged matrix; SynOnly: only the original synteny matrix, not added to 16S; ANI: average nucleotide identity matrix; SyntCov: the synteny coverage matrix added to 16S; and 16S+ANI: ANI added to 16S using the same covariance metric tactic.


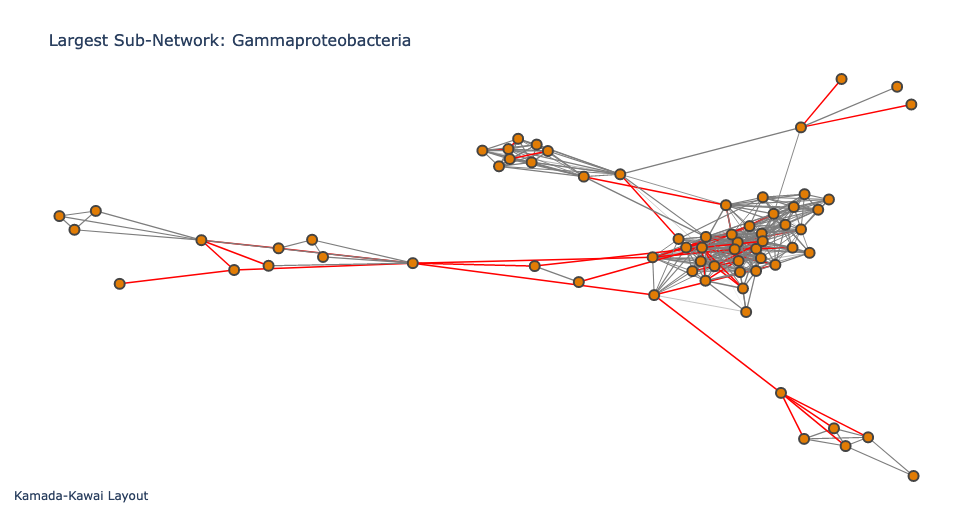


Supplementary Figure 6. Largest sub-network from synteny network (Figure 2). Edges indicate a similarity value present based on synteny. Edge color is determined by weight (synteny similarity). Red edges have weight > 0.9 and edges below are grey. All nodes in the network are part of the class of bacteria, *Gammaproteobacteria.*


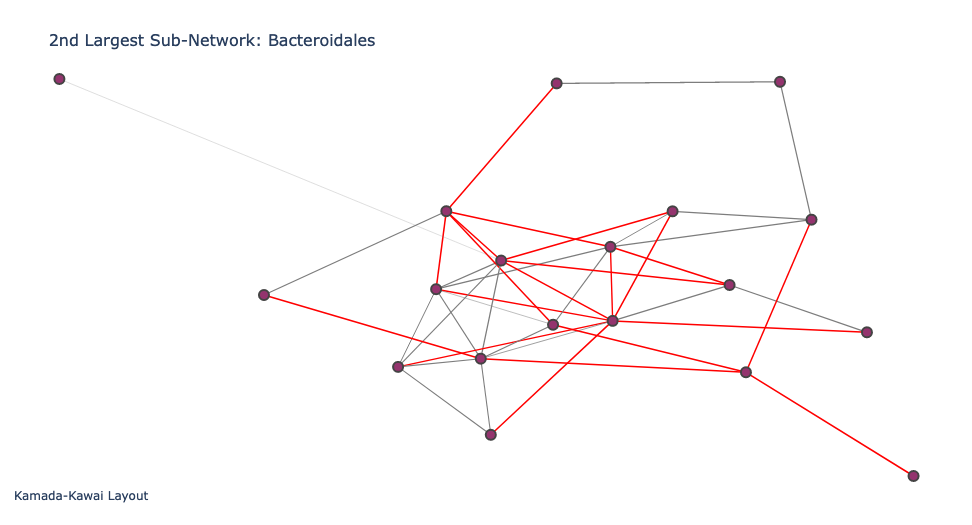


Supplementary Figure 7. Second largest sub-network from synteny network (Figure 2). Edges indicate a similarity value present based on synteny. Edge color is determined by weight (synteny similarity). Red edges have weight > 0.9 and edges below are grey. All nodes in the network are part of the order of bacteria, *Bacteroidales.*


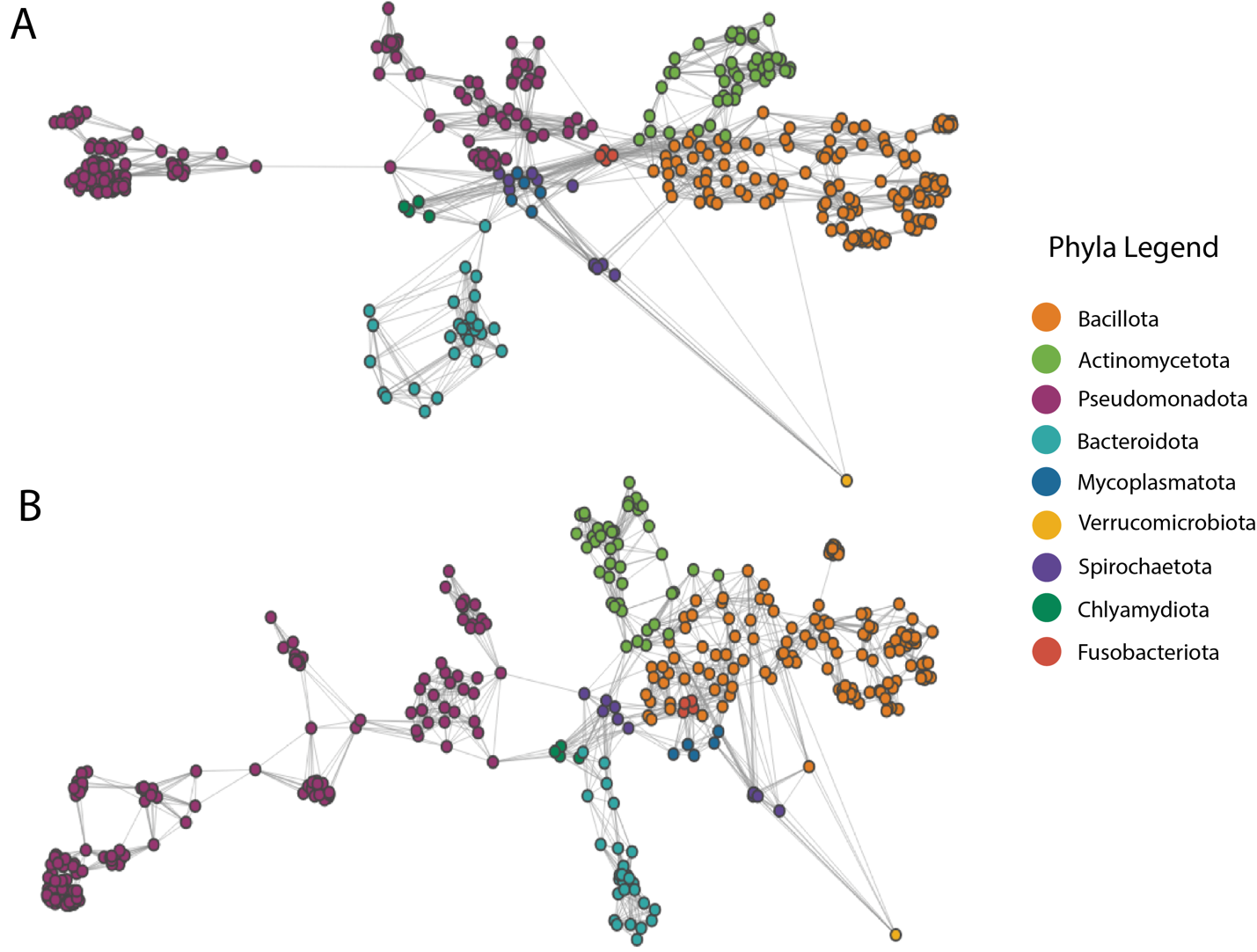


Supplementary Figure 8. Visualizations of k-nearest neighbor graphs for A) 16S rRNA MASH distance data and B) synteny-scaled 16S data. Nodes represent taxa species, which are labeled by taxonomical phyla.


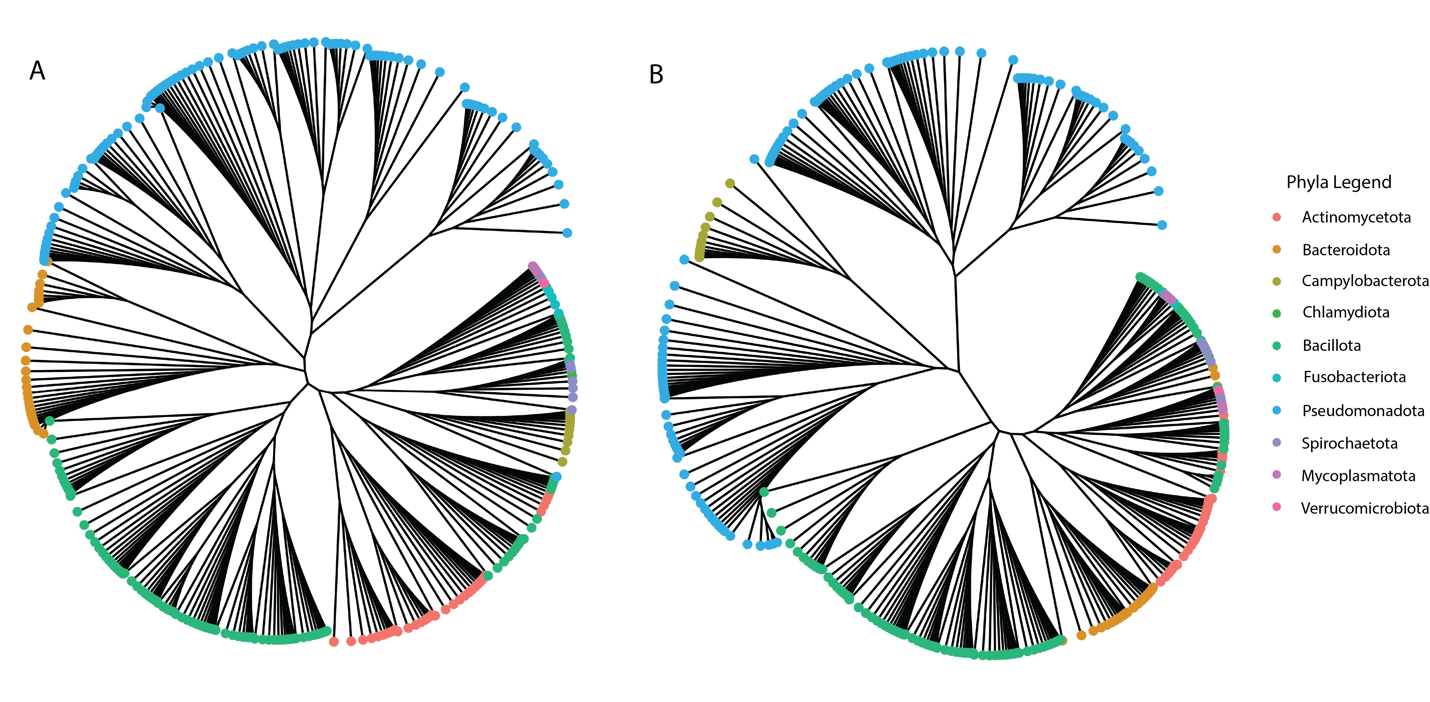
Supplementary Figure 9. Dendrograms based on Girvan-Newman hierarchical clustering, with A) the 16S rRNA distance data and B) the synteny-scaled 16S data. Leaves of the tree branches are labeled with the taxonomical phyla of the species genome.
